# Effects of AT1 receptor antagonism on interstitial and ultrastructural remodeling of heart in response to a hypercaloric diet

**DOI:** 10.14814/phy2.13964

**Published:** 2018-12-27

**Authors:** Silvio A. Oliveira‐Junior, Maeli Dal Pai, Daniele M. Guizoni, Barbara P. Torres, Paula F. Martinez, Dijon H. S. Campos, Marina P. Okoshi, Katashi Okoshi, Carlos R. Padovani, Antonio C. Cicogna

**Affiliations:** ^1^ School of Physical Therapy Federal University of Mato Grosso do Sul Campo Grande Mato Grosso do Sul Brazil; ^2^ Botucatu Biosciences Institute Univ. Estadual Paulista UNESP Botucatu São Paulo Brazil; ^3^ Internal Medicine Department Botucatu Medical School Univ. Estadual Paulista UNESP Botucatu São Paulo Brazil

**Keywords:** Angiotensin, Arterial Pressure, Cardiac Remodeling, Collagen, High‐Fat Diet, Ultrastructure

## Abstract

Palatable hypercaloric feeding has been associated with angiotensin‐II type 1 receptor (AT1R) stimulation and cardiac remodeling. This study analyzed whether AT1R antagonism attenuates cardiac remodeling in rats subjected to a palatable hypercaloric diet. Male Wistar‐Kyoto rats were subjected to a commercial standard rat chow (CD) or a palatable hypercaloric diet (HD) for 35 weeks and then allocated into four groups: CD, CL, HD, and HL; L groups received losartan in drinking water (30 mg/kg/day) for 5 weeks. Body weight, adiposity, and glycemia were evaluated. The cardiovascular study included echocardiography, and myocardial morphometric and ultrastructural evaluation. Myocardial collagen isoforms Type I and III were analyzed by Western blot. Both HD and HL had higher adiposity than their respective controls. Cardiomyocyte cross‐sectional‐area (CD 285 ± 49; HD 344 ± 91; CL 327 ± 49; HL 303 ± 49 μm^2^) and interstitial collagen fractional area were significantly higher in HD than CD and unchanged by losartan. HD showed marked ultrastructural alterations such as myofilament loss, and severe mitochondrial swelling. CL presented higher Type I collagen expression when compared to CD and HL groups. The ultrastructural changes and type I collagen expression were attenuated by losartan in HL. Losartan attenuates systolic dysfunction and ultrastructural abnormalities without changing myocardial interstitial remodeling in rats subjected to a palatable hypercaloric diet.

## Introduction

Left ventricle (LV) remodeling is an adaptive process in response to parietal deformation, and/or multiple stimuli derived from cytokines or growth factors actions that initially enhance cardiac performance and attenuate ventricular wall tension and oxygen demand (Cohn et al. 2000; Swynghedauw et al. 2010). Ventricular remodeling is characterized by time‐dependent changes which include myocardial hypertrophy, extracellular matrix proliferation with variations in types of synthetized collagen, and ultrastructural disturbances (Zile and Brutsaert 2002; Swynghedauw et al. 2010).

In early LV remodeling, the more elastic embryonic Type III collagen is initially deposited; during chronic ventricular remodeling, Type III isoform is replaced by Type I collagen, which has a higher tensile strength (Marijianowski et al. 1995). Characterization of interstitial remodeling of the heart in terms of collagen composition is unclear in experiments with hypercaloric diet interventions. Evidence has shown that hypercaloric diets are strongly associated with cardiac remodeling characterized by morphological and ultrastructural changes in experimental models (Du Toit et al. 2005; Oliveira Junior et al. 2010, 2013; Oliveira‐Junior et al. 2014; Martins et al. 2015). In contrast, Silva et al. (2014) observed reduced Type I collagen expression after 30 weeks of high‐fat diet, while Gonçalves et al. (2016) found increased Type III collagen expression in rats subjected to Western diet for 6 weeks.

Hypercaloric interventions have also been associated with chronic activation of the renin‐angiotensin system (RAS) (Du Toit et al. 2005; Kalupahana and Moustaid‐Moussa 2012; Oliveira‐Junior et al. 2014). Angiotensin II (ANG II) interacts with ANG II Type 1 receptor (AT1R) stimulating myocardial hypertrophy and extracellular matrix proliferation (Cooper et al. 2007; Prabhu and Frangogiannis 2016). Some authors have observed that dietary interventions are associated with RAS activation and cardiac remodeling, which was attenuated by AT1R antagonism (Du Toit et al. 2005; Oliveira‐Junior et al. 2014).

However, we have not identified any studies evaluating whether changes in myocardial collagen Types I and III and ultrastructural disorders are caused by AT1R activation in experimental hypercaloric diets. This investigation was purposed to evaluate the influence of AT1R antagonism on cardiac morphology and interstitial remodeling in rats subjected to a hypercaloric diet with a high fat and carbohydrate content. We tested the hypothesis that hypercaloric diet induces myocardial hypertrophy, ultrastructural disorders, and increases molecular expression of Type I collagen in the heart, which are attenuated by AT1R antagonism.

## Materials and Methods

### Animals and experimental design

Male 60‐day‐old *Wistar‐Kyoto* rats (*n* = 52) were randomly distributed into two groups: control diet (CD) and hypercaloric diet (HD). CD received standard rat chow (3.2 kcal/g) and HD received a hypercaloric diet (4.7 kcal/g) for 30 weeks. Afterwards, animals were assigned to four groups: control (CD); control diet and losartan (CL), hypercaloric diet (HD), and hypercaloric diet and losartan (HL). In addition to their feeding program, CL and HL groups received 30 mg/kg per day of losartan in drinking water for 5 weeks. Age‐matched animals from CD and HD groups continued to receive respective diets for the 5 weeks. Animals were individually housed under controlled conditions of 22–24°C, 50–70% relative humidity, and time‐controlled 12‐h light/dark cycles. All animals had free access to water and chow.

The experimental protocol was approved by our local ethics committee in accordance with Committee on Care and Use of Laboratory Animals (1985).

### Diet composition and general characterization

The hypercaloric diet consisted of five different palatable diets (HD1, HD2, HD3, HD4, and HD5) which were prepared from a mixture of industrialized products and supplementary ingredients added to commercial rat chow. Administration was similar to that in other previous studies (Oliveira Junior et al. 2010, 2013; Oliveira‐Junior et al. 2014).

Food consumption was measured daily and water intake and body weight (BW) were evaluated each week. Weekly calorie intake was calculated as follows: average weekly food consumption × diet energy density. Feed efficiency was determined by: mean BW gain (g)/total calorie intake (Kcal) (Oliveira Junior et al. 2013). Rate of BW gain (%) was calculated as BW gain (%) = [(final BW – initial BW) × 100]/initial BW. At the end of the experimental period, animals were subjected to 12–15 h fasting and anesthetized with sodium pentobarbital (50 mg/kg). BW and body length (nose‐to‐anus length) were used to determine body mass index (BMI) and Lee index. BMI was calculated according to the following equation: BMI = BW (g)/body length^2^ (cm^2^) (Novelli et al. 2007). Lee index was calculated from the following equation: Lee index=cube root of BW (g)/body length (cm) (Bernardis 1970).

At the end of the experiment animals were euthanized by decapitation; thoracotomy was performed to remove and measure adipose depots (AD) from visceral, retroperitoneal, and epidydimal sites. The adiposity index (%) was obtained from the sum of the weights of individual fat pads: ΣAD×100/BW (Oliveira Junior et al. 2010).

### Blood pressure and echocardiography

At the end of the experiment, systolic blood pressure (SBP) was measured by the noninvasive tail‐cuff method with a Narco BioSystems^®^ Electro‐Sphygmomanometer (International Biomedical, Austin, TX) (Cezar et al. 2015). In order to analyze in vivo heart structure and performance, all animals were weighed, anesthetized intramuscularly with a ketamine hydrochloride (50 mg/kg) and xylazine hydrochloride (1 mg/kg) solution, and evaluated via transthoracic echocardiographic examination performed with a commercially available echocardiography machine (HDI 5000 SonoCT Philips, Bothell, WA, USA) equipped with a 12‐MHz phased array transducer. All measurements were obtained by the same observer (KO) as previously described (Lima et al. 2014; Lang et al. 2015; Pagan et al. 2015; Oliveira‐Junior et al. 2017).

### Morphological analyses

The heart was removed at euthanasia. The atria (A) and both right (RV) and left (LV) ventricles were weighed in absolute values and corrected by BW and tibia length. A section from the LV posterior wall (~250–350 μg) was used for histological analysis. Myocardial samples were fixed in 10% formol solution for 48 h and then embedded in paraffin blocks. Histological sections (7 μm) stained with hematoxylin‐eosin (H&E) were used to measure myocyte cell size. Cardiomyocyte cross‐sectional areas were determined for at least 100 cells obtained from 15 to 20 different fields in H&E‐stained sections from each heart (Damatto et al. 2013). Collagen interstitial fraction was determined by Picrosirius red staining of myocardium sections, analyzed under polarized light (Matsubara et al. 1997; Oliveira Junior et al. 2010, 2013). Histological images were obtained using a LEICA DM LS microscope at 40x magnification coupled to a computer equipped with *Image Pro‐plus*, an image analysis program (Media Cybernetics, Silver Spring, Maryland, USA).

Four rats from each group were used for the ultrastructural study. Small fragments of LV papillary muscle were fixed in Karnovsky's fixative (0.12 mol/L phosphate, pH 7.2) for 1 to 2 h followed by postfixation in 1% osmium tetroxide in 0.1 mol/L phosphate buffer for 2 h (Oliveira Junior et al. 2010). After dehydration in a graded ethanol series, fragments were embedded in epoxy resin. Ultrathin sections were double‐stained with uranyl acetate and lead citrate and examined under electron microscope (Phillips EM 301).

### Western‐blotting analysis

The expression of myocardial collagen isoforms I and III was analyzed by Western blot. Sample preparation methods and electrophoresis conditions are detailed in previous studies (Silva et al. 2014; Guizoni et al. 2016; Martinez et al. 2016). Western blot analysis was performed using antibodies against collagen types I and III (1:10000; ABCAM, UK, Cambridge). Protein levels were normalized to those of β‐actin or GAPDH (1:1000; Santa Cruz Biotechnology, Inc., Santa Cruz, CA).

### Statistical Analyses

Results are expressed as descriptive measures of centralization and variability. Taking into account a design consisting of diet and pharmacological intervention, parameters were analyzed by two‐way ANOVA for independent groups. When significant differences were found (*P* < 0.05), a post hoc Tukey's multiple comparisons test for parametric, or a Dunn's test for nonparametric distributions was performed. Level of significance was considered at 5%.

## Results

Hypercaloric diet was associated with increased calorie intake, feed efficiency, body mass index, BW gain, adiposity, and Lee index in both HD and HL groups, when compared to their respective controls. AT1R antagonism did not affect these results. BW was not changed by diet or losartan (Table 1).

**Table 1:** General data

Variable	Groups			
	CD	HD	CL	HL
Calorie intake (Kcal/g)	87.0 ± 6.6	70.9 ± 7.3*	87.8 ± 7.1	72.9 ± 8.0‡
F. efficiency (mg/Kcal)	13.8 ± 1.8	17.9 ± 3.3*	13.9 ± 1.8	17.4 ± 2.6‡
Body weight (g)	582 ± 57	616 ± 70	576 ± 60	622 ± 74
BMI (g/cm^2^)	0.73 ± 0.06	0.79 ± 0.09*	0.73 ± 0.06	0.80 ± 0.07 ‡
Body mass gain (%)	84.3 ± 13.8	101.3 ± 29.6*	85.9 ± 14.3	102.4 ± 20.8 ‡
Adiposity (%)	7.11 ± 2.27	10.64 ± 3.29*	7.14 ± 1.75	10.38 ± 2.70‡
Lee index (g/cm^3^)	6.62 ± 0.63	7.19 ± 0.77*	6.56 ± 0.60	7.17 ± 0.72‡
Glucose (mg/dL)	79.2 ± 7.7	81.6 ± 7.3	81.2 ± 8.3	85.5 ± 8.2

Values expressed as mean ± SD; *n* = 13 animals per group.

F. efficiency, feed efficiency; BMI, body mass index

*P < 0.05 versus CD; ^†^ *P* < 0.05 versus HD; ^‡^ *P* < 0.05 versus CL; *Two‐Way* ANOVA and Tukey's test.

**Figure 1:**
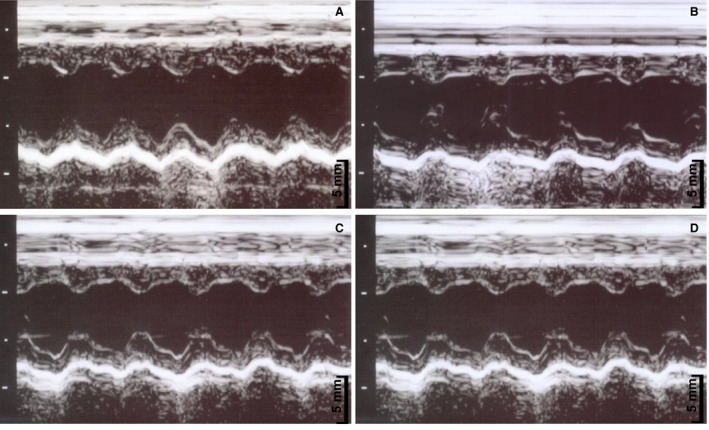
Representative left ventricular M‐mode echocardiographic images according to groups; (A) CD, control diet; (B) HD, hypercaloric diet; (C) CL, control diet treated with losartan; (D) HL, hypercaloric diet treated with losartan.

Diet intervention did not change SBP and ventricular size. Losartan reduced SBP and increased heart rate in both medicated groups. AT1R antagonism decreased posterior wall diastolic thickness (PWT) in CL compared to CD. HD had higher ejection fraction than CD; this difference was not observed between the CL and HL groups (Table 2). Representative figures of M‐mode pictures according to group are presented in the Figure 1.

**Table 2:** Blood pressure and echocardiographic parameters

Variable	Groups			
	CD	HD	CL	HL
SBP (mmHg)	120 ± 10	121 ± 12	111 ± 10*	110 ± 14†
Heart rate (beats/min)	258 ± 57	272 ± 55	299 ± 47*	321 ± 35†
LA/AO	1.42 ± 0.10	1.37 ± 0.08	1.32 ± 0.10	1.38 ± 0.12
LVEDd (mm)	8.44 ± 0.63	8.11 ± 0.44	8.22 ± 0.25	8.21 ± 0.48
LVESd (mm)	4.29 ± 0.39	4.24 ± 0.31	4.32 ± 0.37	4.08 ± 0.41
PWT (mm)	1.57 ± 0.06	1.55 ± 0.07	1.50 ± 0.09 *	1.51 ± 0.07
LV relative thickness	0.38 ± 0.02	0.37 ± 0.02	0.36 ± 0.02	0.37 ± 0.03
EFS (%)	48.5 ± 4.0	48.6 ± 3.3	48.5 ± 3.0	51.2 ± 4.1
Ejection fraction (%)	83.9 ± 2.1	86.9 ± 3.2 *	83.8 ± 3.4	85.5 ± 3.4
PWSV (mm/sec)	36.8 ± 3.3	38.6 ± 4.6	37.0 ± 4.2	37.6 ± 3.5

Values expressed as mean ± SD.

SBP, systolic blood pressure; LA/AO, ratio between diameters of the left atrium and aorta; LVEDd, left ventricular (LV) end‐diastolic diameter; LVESd, LV end‐systolic diameter; PWT: LV posterior wall diastolic thickness; LV relative thickness, relation between LV posterior wall systolic thickness and LVEDd; EFS, endocardial fractional shortening; PWSV, posterior wall shortening velocity.

*P < 0.05 versus CD; ^†^ *P* < 0.05 versus HD; *Two‐Way* ANOVA and Tukey's test.

Macroscopic morphology was not changed by diet or losartan administration (Table 3). Hypercaloric diet increased cardiomyocyte cross‐sectional area (*P* = 0.026) and interstitial collagen fractional area in HD compared to CD group. Losartan did not change these results as CL and HL had similar responses in terms of histological measurements (Table 3).

**Table 3:** Cardiac anatomic and morphometric parameters

Variable	Groups			
	CD	HD	CL	HL
Atria weight (g)	0.113 ± 0.010	0.111 ± 0.017	0.105 ± 0.011	0.105 ± 0.023
AW/BW (mg/g)	0.203 ± 0.020	0.183 ± 0.016	0.192 ± 0.020	0.173 ± 0.024
AW/tibia (g/m)	2.45 ± 0.19	2.40 ± 0.37	2.27 ± 0.18	2.29 ± 0.47
RV weight (g)	0.27 ± 0.03	0.27 ± 0.03	0.25 ± 0.02	0.26 ± 0.03
RVW/BW (mg/g)	0.49 ± 0.03	0.45 ± 0.07	0.45 ± 0.05	0.43 ± 0.03
RVW/tibia (g/m)	5.91 ± 0.57	5.76 ± 0.53	5.28 ± 0.48	5.64 ± 0.71
LV weight (g)	0.87 ± 0.09	0.86 ± 0.09	0.81 ± 0.06	0.77 ± 0.07
LVW/BW (mg/g)	1.56 ± 0.07	1.43 ± 0.10	1.48 ± 0.11	1.30 ± 0.10
LVW/tibia (g/m)	17.5 ± 5.44	18.7 ± 1.85	17.5 ± 1.13	16.9 ± 1.38
CSA (μm^2^)	285 ± 49	344 ± 91*	327 ± 49	303 ± 49
CIF (%)	2.48 ± 0.85	4.13 ± 1.41*	2.21 ± 0.66	4.31 ± 1.19‡

Values expressed as mean ± SD.

AW/BW, ratio between atria and body weights; AW/tibia, ratio between atria weight and tibia length; RV weight, right ventricle weight; RVW/BW, ratio between right ventricle and body weights; RVW/tibia, ratio between atria weight and tibia length; LV weight, left ventricle weight; LVW/BW, ratio between left ventricle (LV) and body weights; LVW/Tibia, ratio between LV weight and tibia length; CSA, cross‐sectional area of cardiomyocytes; CIF, collagen interstitial fraction

*P < 0.05 versus CD; ^‡^ *P* < 0.05 versus CL; *Two‐Way* ANOVA and Tukey's test.

**Figure 2:**
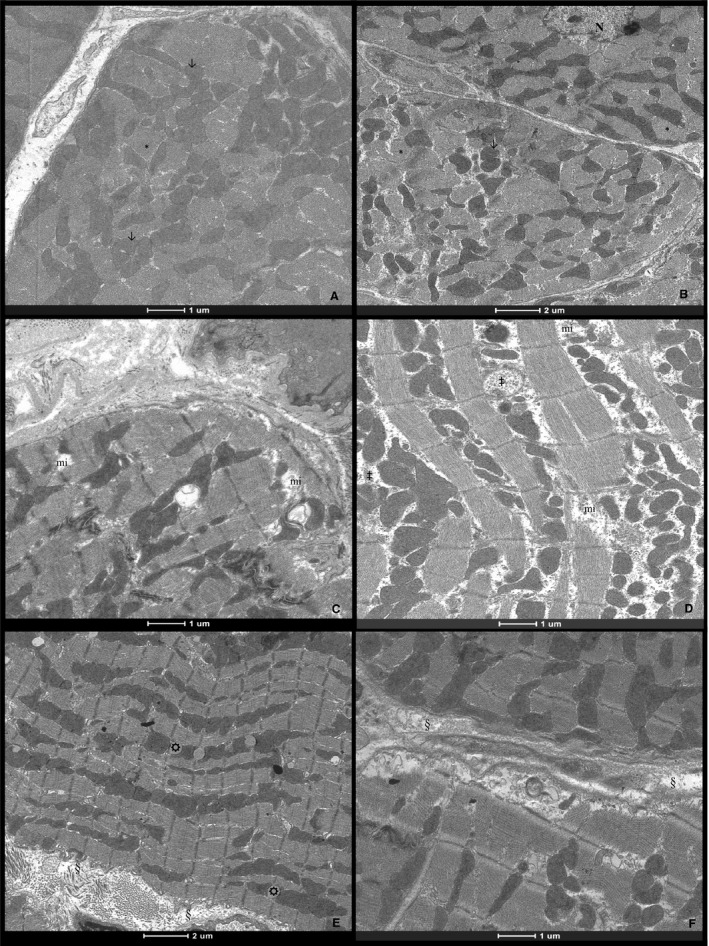
Cardiac striated muscle. (A) and (B) electron micrographs of cardiomyocytes from control diet (CD) and losartan treated control diet (CL) groups, respectively. Muscle fibers with normal aspect in A and B. Myofibrils (*); Mitochondria (arrow); Nucleous (N). (C) and (D) electron micrographs of cardiomyocytes from hypercaloric diet (HD) group. Myofilaments and Z‐line disorganization of sarcomeres and myofibril loss (mi) in C and D. Swelling mitochondria with disorganized or absence of cristae (&) in D. (E) and (F) electron micrographs of cardiomyocytes from the losartan treated hypercaloric diet group (HL). Intracellular lipid droplets (☼) in E; myofibril reorganization (§) in E and F.

Ultrastructural evaluation showed that CD and CL groups had normal cardiomyocyte morphology, including sarcolemma, myofibrils, mitochondria, central nucleus, and sarcoplasmic reticulum (Fig. 2A and B). HD cardiomyocytes had extensive alterations characterized by Z‐line disorganization, swelling, severe mitochondria degradation and polymorphisms with disorganized or absent cristae, and lipid droplets. Many cells presented areas with myofilament loss and mitochondria swelling (Fig. 2C and D). Losartan attenuated these alterations in HL, which had lower cellular degradation and important areas of myofibril reorganization (Fig. 2E and F).

**Figure 3:**
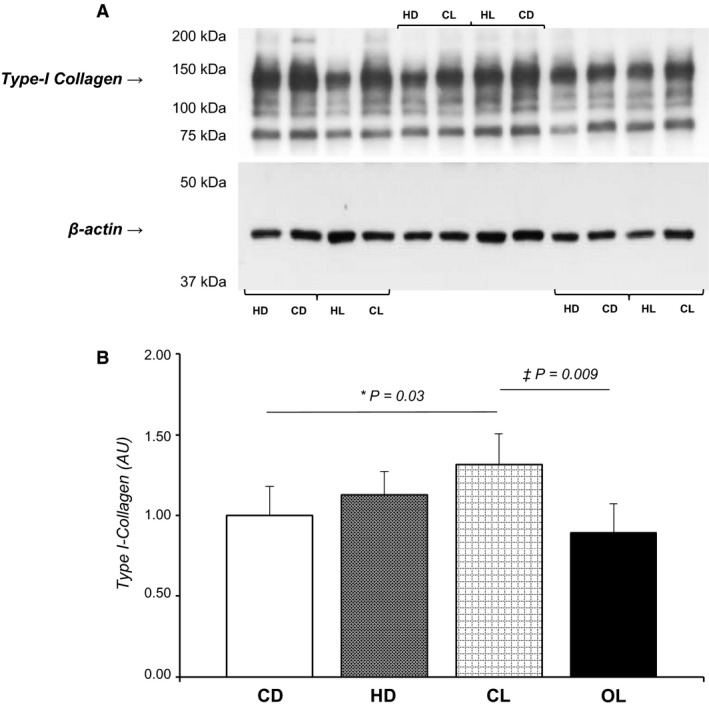
Protein expression of Type I collagen in myocardium. Values presented as mean ± SD. Protein levels were normalized to β‐actin levels; (A) Protein bands of Type I collagen and β‐actin levels; CD (*n* = 6), control diet; HD (*n* = 6), hypercaloric diet; CL (*n* = 6), control diet treated with losartan; HL (*n* = 6), hypercaloric diet treated with losartan. (B) Type I collagen expression values according to two sources of variation: effects of diet and medication; *P < 0.05 versus CD; &*P* < 0.05 versus CL; *Two‐Way* ANOVA and Tukey's test.

**Figure 4:**
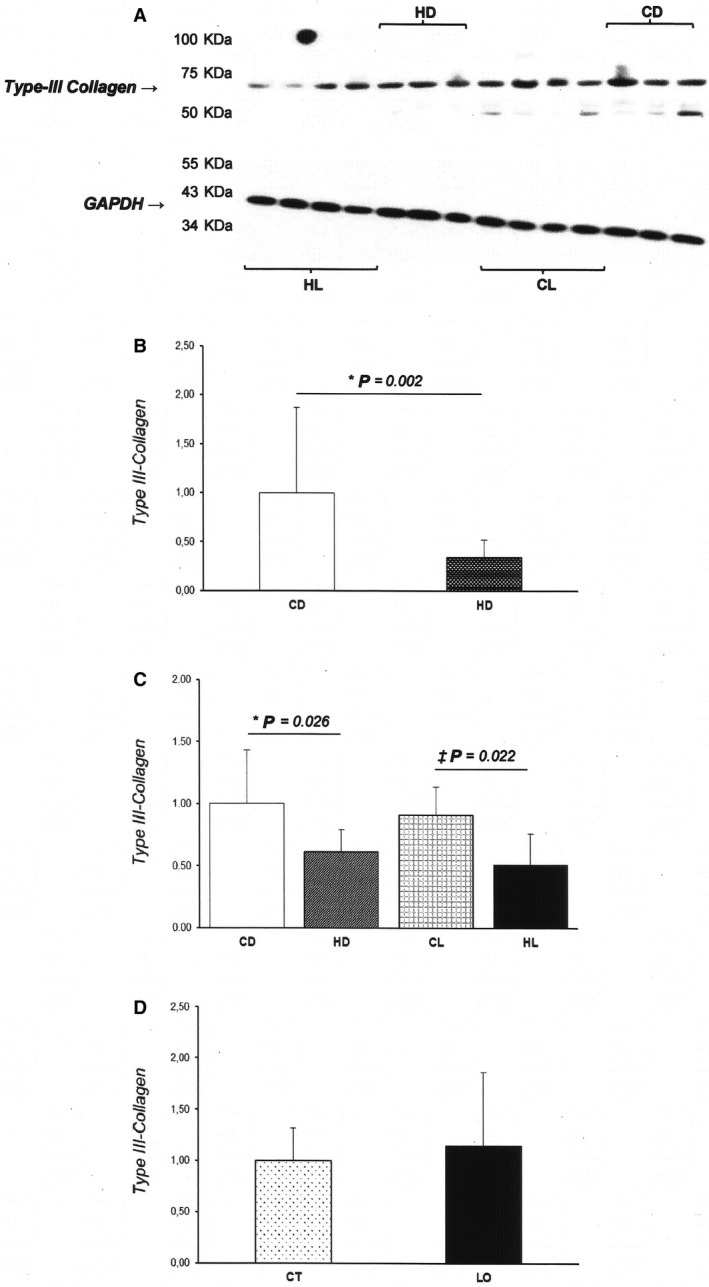
Protein levels of Type III collagen in myocardium. Values presented as mean ± SD. Protein levels were normalized to GAPDH levels; (A) Protein bands of Type III collagen and GAPDH levels; CD (*n* = 6), control diet; HD (*n* = 6), hypercaloric diet; CL (*n* = 6), control diet treated with losartan; HL (*n* = 6), hypercaloric diet treated with losartan. (B) Type III collagen expression values according to one source of variation: effects of diet; CD, control diet; HD, hypercaloric diet; * *P* < 0.05 versus CD. (C) Type III collagen expression values according to two sources of variation: effects of diet and medication; * *P* < 0.05 versus CD; &*P* < 0.05 versus CL. (D) Type III collagen expression values according to one source of variation: effects of medication; CT, control; LO, Losartan. *Two‐Way* ANOVA and Tukey's test.

We observed a significant interaction between diet and drug for Type I collagen expression (*P* = 0.016; Fig. 3). While CD and HD showed similar results, CL presented higher Type I collagen expression when compared to CD and HL groups (CD: 1.00 ± 0.32; HD: 1.13 ± 0.29; CL: 1.32 ± 0.20; HL: 0.90 ± 0.35 AU; Fig. 3B). On the other hand, dietary intervention, *per se*, reduced Type III collagen expression in HD and HL groups when compared to their respective control groups (CD: 1.00 ± 0.43, HD: 0.61 ± 0.18 AU, *P* < 0.05; CL: 0.91 ± 0.23, HL: 0.51 ± 0.25 AU, *P* < 0.05; Fig. 4B).

## Discussion

This study evaluated the impact of AT1R antagonism on LV morphology and interstitial remodeling in rats subjected to a hypercaloric diet. In accordance with our hypothesis, hypercaloric diet promoted myocardial hypertrophy and interstitial fibrosis, and important ultrastructural changes in HD group. These changes were combined with improved systolic performance and reduced myocardial expression of Type III collagen. Losartan normalized morphological alterations and reduced the expression of Type I collagen with no influence on interstitial collagen fraction in the HL group.

Diet composition was based on experimental models with cafeteria diet, which is characterized by a high energy content, different tastes and shapes, being therefore closer to food generally consumed by humans (Nascimento et al. 2008). Hypercaloric diet was obtained by adding a combination of industrialized products to commercial rat chow; this has been associated with high fatty acid content combined with sucrose overload. As a consequence, dietary intervention promoted higher body weight gain and adiposity based on increased feeding efficiency (Table 1). These results are similar to those seen in previous studies (Oliveira Junior et al. 2013; Oliveira‐Junior et al. 2014). The current diet effects were observed in both losartan groups, as it did not affect body composition and nutritional findings.

Systolic blood pressure was not affected by the hypercaloric diet. Several studies have shown that dietary obesity is accompanied by vascular hyperactivity, an important mechanism in the onset of arterial hypertension (Bourgoin et al. 2008; Nascimento et al. 2011). Hypercaloric and high‐fat diets have been associated with high blood pressure values at night, when rodents are behaviorally active; also, these animals commonly present unchanged resting blood pressure values (Sedová et al. 2004). On the other hand, increased nitric oxide bioavailability in rats submitted to a high‐fat diet could represent an adaptive mechanism which counteracts the detrimental impact of hypercaloric diet on vascular reactivity (Plotnick et al. 1997; Sato et al. 2002). This improvement in the endothelial nitric oxide pathway could also explain the absence of changes in blood pressure between different dietary groups. Despite of this, it is not discarded that high pressure levels could be contributing for morphological and molecular adaptive changes supported by hypercaloric diet intervention.

The HD group had myocardial hypertrophy and interstitial remodeling. AT1R antagonism normalized cardiomyocyte hypertrophy, showing that activation of the renin‐angiotensin system contributes to cardiac remodeling in response to hypercaloric diet. Importantly, HL presented higher interstitial collagen fraction than CL, but losartan reduced Type I collagen expression in HL.

Myocardial hypertrophy results from an imbalance between stimulating and inhibiting molecular agents that also affect extracellular matrix proliferation (Cohn et al. 2000; Swynghedauw et al. 2010). RAS, particularly ANG II, plays a major role in the pathogenesis of cardiac fibrosis (Huang et al. 2010; Iwata et al. 2011) and hypertrophy. ANG II increases collagen proliferation and expression, and migration of cardiac fibroblasts by activating several cell signaling pathways such as transforming growth factor β (TGF‐β) and mitogen‐activated protein kinases (MAPKs) pathways (Olson et al. 2005, 2008; Lijnen et al. 2012). Li et al. (2015) showed that ANG II stimulates collagen I expression in a dose‐ and time‐dependent manner in cardiac fibroblasts, confirming the fact that AT1R antagonism reduced Type I collagen synthesis in the HL group.

The myocardial extracellular matrix (ECM) plays a pivotal role in cardiac remodeling caused by excessive activation of the sympathetic nervous system (SNS) and renin–angiotensin–aldosterone system (RAAS) (Spinale et al. 2002). The enzyme system primarily responsible for ECM turnover is the matrix metalloproteinases (MMPs), which can be blocked by tissue inhibitors of the MMPs (TIMPs) (Yang et al. 2009). Increased MMP activity and/or increased MMP–TIMP complex formation accelerates myocardial remodeling (Webb et al. 2006; Zheng et al. 2016). After AT1R blockade, it is possible that other agents, such as growth factors and dietary effects are associated with collagen degradation. Although controversial the association between leptin and myocardial collagen Type I, such as increased expression (Madani et al. 2006; Schram et al. 2008, 2010) and decreased synthesis (Schram et al. 2008) of procollagen, there is agreement that leptin increases the activity of metalloproteinase (MMP)‐2 and mRNA expression of MMP‐9 (Madani et al. 2006; Schroeter et al. 2012), participants in myocardial Type I collagen degradation. It is therefore possible that increased MMP‐2 and MMP‐9 activity is responsible for the reduction in myocardial Type I collagen.

Considering the dietary effects, Földes et al. (2006) observed that saturated or unsaturated fat‐rich diets induced activation of the peptides involved in cardiac remodeling: activator protein‐1 (AP‐1) and mitogen‐activated protein kinases (MAPKs). Their effects include increased interstitial fibrosis through TGF‐β. Also, lipotoxicity (Butler et al. 2017) and oxidative stress (Yu et al. 2018) are not disregarded in the interstitial response. Recently, Glenn et al. (2015) demonstrated that excessive lipid accumulation within cardiomyocytes amplifies the fibrotic effects of ANG II.

The HD group had a marked occurrence of intracellular lipid droplets in LV. Lipid accumulation in cardiomyocytes has also been described in doxorubicin‐induced cardiomyopathy (Zimmerman et al. 1994), spontaneously hypertensive rats submitted to hypercaloric diet (Oliveira Junior et al. 2010) and in both ischemic and nonischemic myocardium after cardiac ischemia (Greve et al. 1990; Saito et al. 2013). After myocardium damage, normal fatty acid oxidation for energy production in cardiomyocytes is suppressed or impaired, and lipid droplets accumulate. Lipid droplets in cardiomyocytes occur after inhibition of fatty acid metabolism (Schulze et al. 2016). Ultrastructural evidence of lipid droplet accumulation has also been observed in obese patients and in the acute phase of several heart diseases (Greve et al. 1990; Saito et al. 2013). Therefore, lipid droplet accumulation can induce cardiac lipotoxicity and additional myocardial damage.

Other ultrastructural changes in the HD group were myofilament loss and disorganization, and severe mitochondrial swelling cristae disorder or absence. Disorganization and lack of myofibrils, myofilaments and Z discs, and disconnection between myocytes can hamper the coordinated transmission of muscular contraction and reduce myocardial performance (Sugizaki et al. 2005). It is noteworthy that losartan attenuated these morphological alterations in the HL group. Adipose tissue hypertrophy due to hypercaloric intervention has been associated with RAAS up‐regulation and the production of inflammatory cytokines which may jeopardize cardiac tissue (Kalupahana and Moustaid‐Moussa 2012). Therefore, our results support the fact that hypercaloric diet‐induced cardiac remodeling is associated with angiotensin aldosterone‐rennin system activation.

In conclusion, AT1R antagonism treatment attenuates systolic dysfunction and ultrastructural abnormalities without changing myocardial interstitial remodeling in rats subjected to a palatable hypercaloric diet.

## Conflict of Interest

The authors declare that there are no potential conflicts of interest relevant to this article.
